# Supplementary material for: Decisions are expedited through multiple neural adjustments spanning the sensorimotor hierarchy
Source: Nat Commun. 2018 Sep 7;9:3627. doi: 10.1038/s41467-018-06117-0 (PMC6128824; doi:10.1038/s41467-018-06117-0)
Supplement: Supplementary file 1 — Supplementary Information [file 41467_2018_6117_MOESM1_ESM.pdf]

Supplementary Information

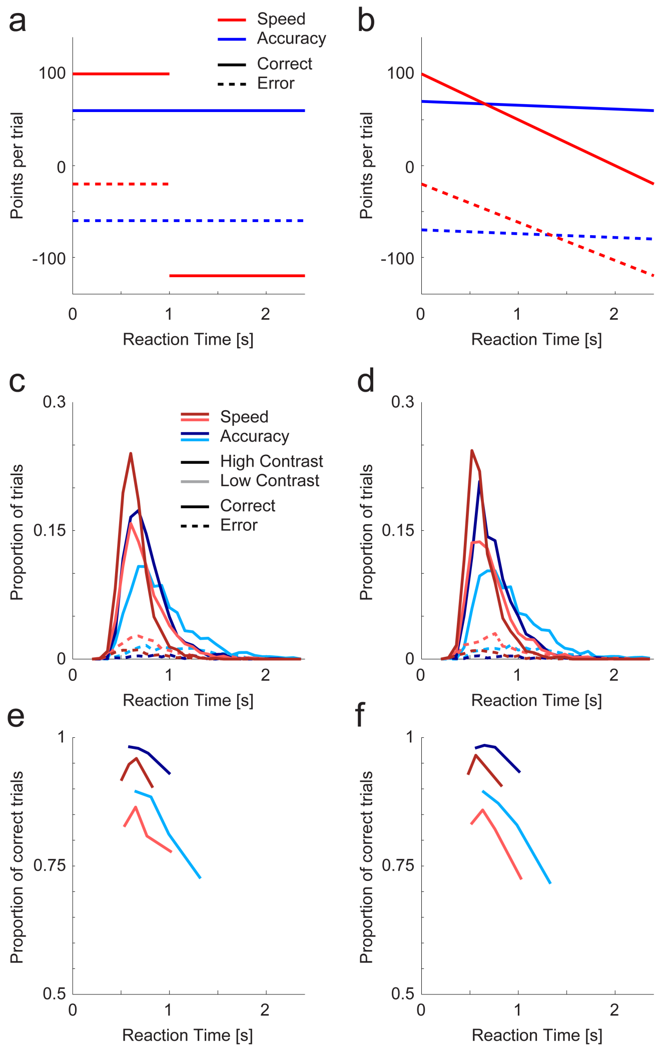

Supplementary Figure 1: Reward structures and behavioral impact of Speed/Accuracy emphasis

In different blocks, Speed/Accuracy emphasis was encouraged either by (a) awarding points for correct responses (Speed: 100 pts, vs. loss of 20 pts for errors, Accuracy: 60 pts vs. -60 pts) within a discrete deadline (1s in Speed vs. 2.4s in Accuracy, loss of 120 pts/60 pts for any later response), or (b) by adjusting a declining slope of points awarded as a function of reaction time (Speed: -50 pts/s vs. Accuracy: -4.2 pts/s). Solid lines indicate the number of points won for a correct response, while dashed lines indicate the penalty for incorrect responses. In both types of regime manipulation, Speed and Accuracy trials were randomly interleaved for comparison against one another. If no response was recorded before stimulus offset (2.4s), the maximum penalty of the respective reward regime was awarded. This occurred on an extremely low proportion of trials (0.0164 ± 0.011 across all subjects and experimental conditions). The proportion of such missed trials was highest for low contrast trials under Accuracy emphasis

(0.0035 $\pm$ 0.0081; Speed pressure, low Contrast: 0.00080 $\pm$ 0.0023; Accuracy emphasis, high Contrast: 0.00027 $\pm$ 0.0011; Speed pressure, high Contrast: 0.00080 $\pm$ 0.0017). To motivate participants, they were informed that their monetary reward for participation in the experiment would be a function of the points won on four randomly chosen experimental blocks.
(c-d) Reaction time distributions of correct (solid) and error (dashed) trials were not significantly different between response time constraints implemented through deadlines (c) and linearly decreasing rewards over reaction time (d) between the two speed or between the two accuracy conditions (Kolmogorov-Smirnov, all $p>0.7$).
*(e-f) Conditional accuracy functions of both methods of response time constraints - deadlines (e) and decreasing rewards over reaction time (f) - show the characteristic decrease in response accuracy over reaction time in combination with low conditional accuracy for the fastest responses under speed pressure. Analyses of accuracy and reaction times revealed that the methods were equally effective in altering decision speed and accuracy, producing the same qualitative pattern of effects (see c-f). We therefore collapsed across the deadline and decreasing-reward conditions in all analyses. Subjects won significantly more points on Speed trials than on Accuracy trials (2-Way ANOVA: $F(1,15) = 68.8$; $p = 5.48*10^{-7}$; Supplementary Table **5h**).*

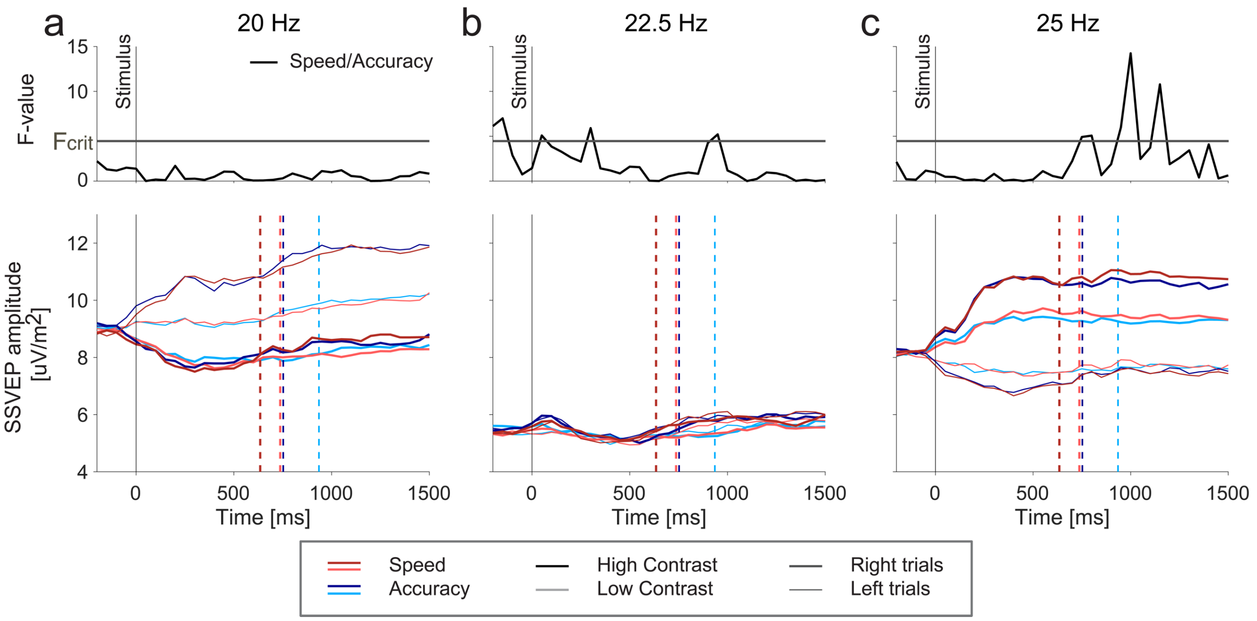

Supplementary Figure 2: Spectral traces of individual SSVEP frequencies

SSVEP traces for individual phase-reversal frequencies without normalization or baseline correction revealed that the boost in differential SSVEP during decision formation under the Speed regime was unlikely driven by a general boost in both SSVEP frequencies, either before or during decision formation. Bottom plots in panels a, b and c trace the time courses of the 20, 22.5 and 25 Hz frequency bins respectively. Dashed vertical lines indicate mean RT for each condition. F-value time-courses (top panels) illustrate the lack of a main effect of Speed/Accuracy regime during the decision formation period even though both individual SSVEP frequencies (a+c) are strongly modulated by the contrast of the respective visual target. An intermediate frequency (b) is shown as a control, to verify the lack of modulation of non-excited frequencies and lack of effect of Speed/Accuracy regime on frequencies neighboring the sensory-driven SSVEPs.

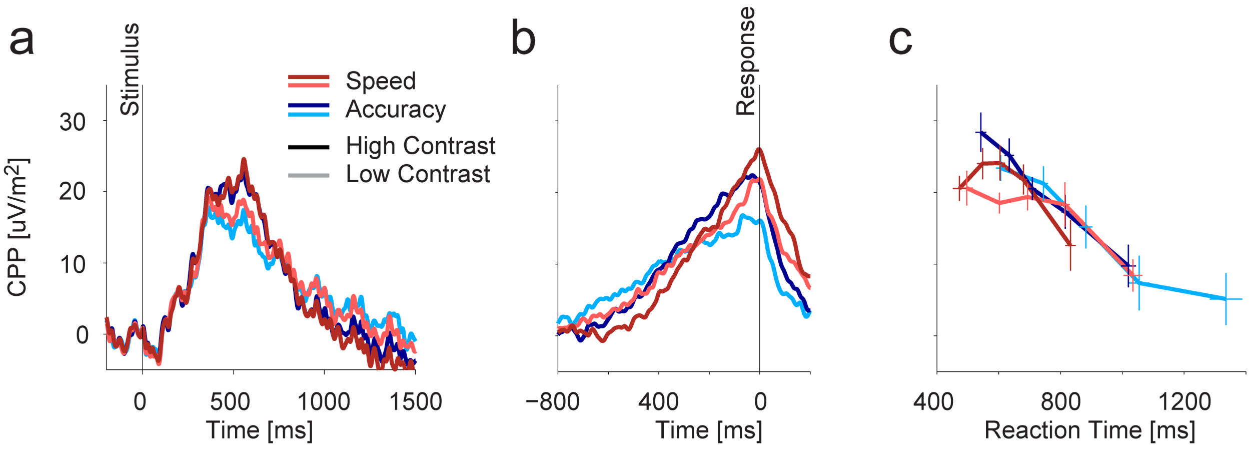

Supplementary Figure 3: Centro-parietal positivity without subtracting stimulus-locked auditory evoked response. *All analyses in the main body of the article concerning the centro-parietal positivity were carried out on waveforms from which an ERP component evoked by an auditory stimulus played at evidence onset was subtracted out (see Methods). Here we provide an analysis of the original data without such auditory EP subtraction to verify that the effects on CPP reported in the main text could not be attributed to such a technicality of the analysis. CPP amplitude at pre-stimulus baseline was measured in regime cue-locked epochs and therefore not influenced by this processing step. (a) Stimulus-locked and (b) Response-locked CPP traces, the latter revealing that the slope of the CPP maintained its significant relationships with Speed/Accuracy regime (F(1,15) = 11.6, p = 0.0039; Supplementary Table **5i**) and Contrast (F(1,15) = 21.0, p=0.00036). (c) The amplitude of the CPP at response was significantly increased for faster reaction time* ($\chi^2(1)$ = 23.2, p = $1.45*10^{-6}$, Supplementary Statistical analysis details 1k*) and under Accuracy compared to Speed pressure* ($\chi^2(1)$ = 4.2, p = 0.040, *Supplementary Table **3b**), verifying the reliability of the effects reported for the data with subtracted auditory activation.*

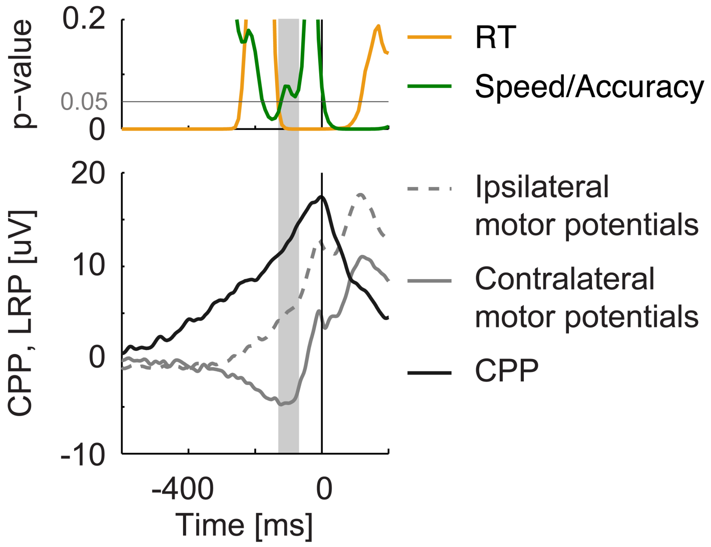

Supplementary Figure 4: Unilateral motor potentials and determination of CPP measurement window. *Unilateral motor potentials measured at lateral sites over motor cortex (standard sites 'C3' and 'C4') locked to response execution. As is typical, the motor signal contralateral to the response-executing limb initially exhibits a slow negativity known as the Readiness Potential, before reversing in polarity just before the response[1]. In the present data this inflection point occurred approximately 100 ms before the button click (lower panel). Based on previous work interpreting the inflection point of effector-selective activity as the time of decision commitment[2], and evidence from the monkey saccade system that commitment does occur some tens of milliseconds before action onset[3], we calculated the amplitude of the centro-parietal positivity "at response" as the integrated amplitude in a 60 ms-window centered on this timepoint (gray shading). This, however, implies the assumption that there are no delays in the transmission of feed-forward evidence-accumulation signals into the motor preparation and response circuitry. To understand our results with respect to this uncertainty regarding such time lags between decision commitment and the registration of the button click, we traced the size of the principal effects of interest on CPP amplitude as a function of the time of measuring "CPP at decision commitment". Specifically, we repeated the same linear mixed-effects models on the CPP amplitude as in the main text, in several 60-ms time windows centered on the time points indicated on the abscissa. In the upper panel the p-values for the effect of Speed/Accuracy Regime are shown, with the gray horizontal line indicating our alpha-level of 0.05. While the significant effect of reaction time on CPP amplitude at response is stable over different time windows of measurement, time windows earlier than the one chosen in the main text (the 60-ms window centered around the mean motor time of -100 ms), indicate a significant effect of speed pressure on CPP amplitude at response ($p < 0.05$ for windows centered anywhere between -170 to -130 ms).*

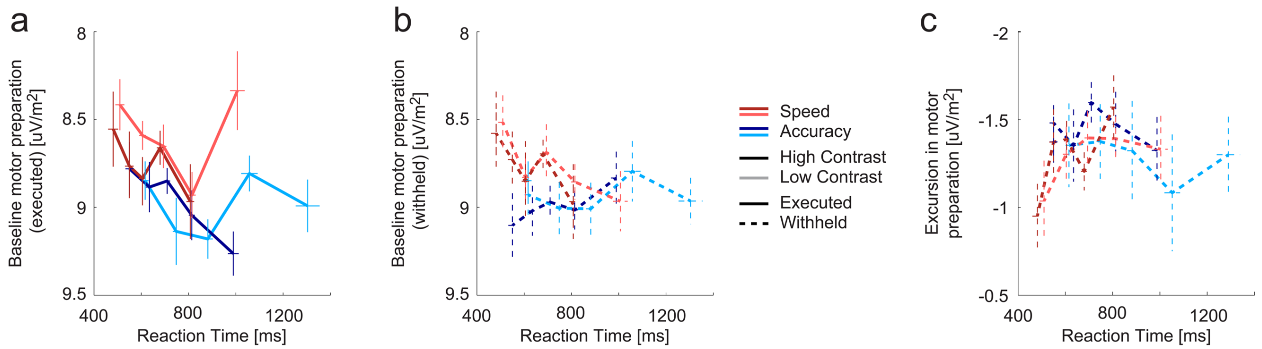

Supplementary Figure 5: Motor preparation as a function of reaction time

*(a) Baseline motor preparation was measured as the mean Mu/Beta amplitude (8-30Hz) in the 300ms before stimulus onset. Baseline motor preparation for the ultimately executed response was relatively increased under speed pressure and decreased for later reaction time (see main text). (b) Motor preparation at baseline for the withheld response showed the same relationship with RT. Such a predictive relationship constitutes empirical evidence for starting point variability across trials at the motor level. The fact that the motor preparation for both the ultimately executed and the withheld response show such correlations suggests that these trial-by-trial fluctuations may reflect fluctuations in subjective speed emphasis across trials. We also observed a significant correlation between the differential motor preparation at baseline and RT (linear mixed-effect model; $\chi^2(1) = 4.6$, $p = 0.031$, Supplementary Table **3c**).*

(c) The excursion in motor preparation for the withheld response was measured as ipsilateral Mu/Beta amplitude just before evidence onset subtracted from the Mu/Beta amplitude ipsilateral to the responding hand at the time of response. As this analysis was aimed at examining the time-dependence in the evidence-independent build-up of motor preparation, we only included trials that resulted in a correct response, so that the sensory evidence actually opposed these ipsilateral signals reflecting partially prepared, but ultimately withheld responses.

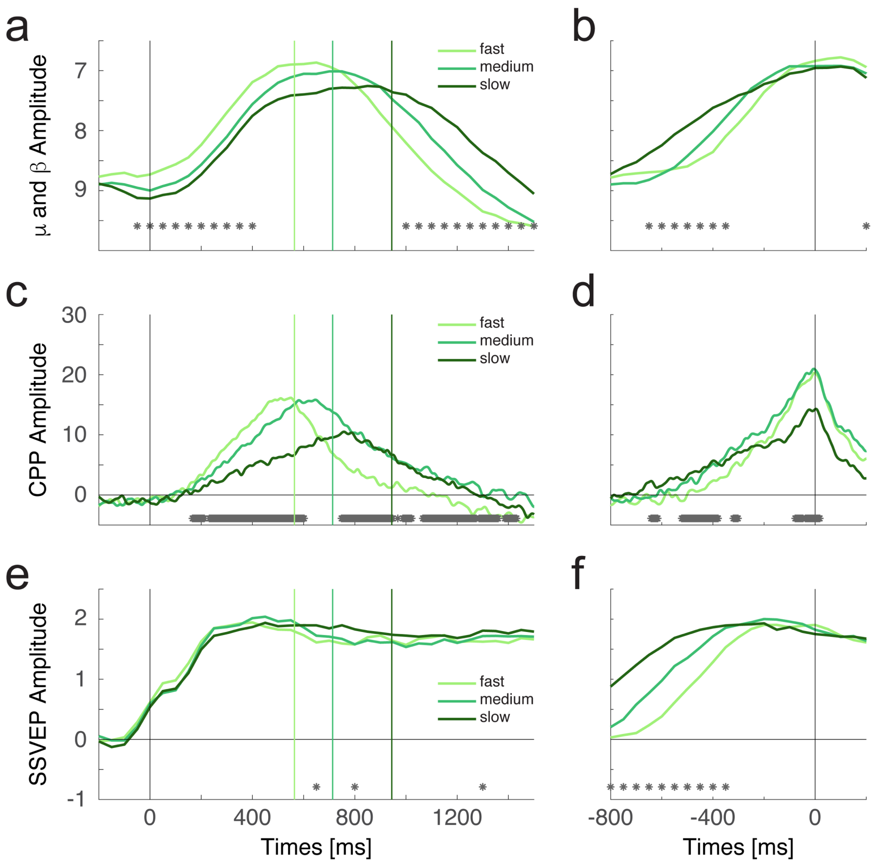

Supplementary Figure 6: Time course of sensory encoding, accumulation and motor preparation as a function of RT

For each individual subject and condition (Regime, Contrast, onset delay and Target Type), trials were split into reaction time tertiles and each tertile was then collapsed across conditions. Mean neural signals of the three RT bins are plotted locked to evidence onset (a, c, e) and response (b, d, f). Vertical lines in the stimulus-locked panels indicate mean RT per tertile. For each time point, we computed the correlation between RT (tertile means) and mean signal amplitude within subjects. We then computed t-tests to test whether the distributions were significantly different from zero across subjects. Time points with significant correlations are marked with gray stars at the bottom of each panel. (a) Stimulus-locked Mu/Beta signals predict reaction time shortly before and after evidence onset with greater motor preparation (lower Mu/Beta amplitude) predicting faster response times. Significant negative correlations between motor preparation and RT more than 1000ms after evidence onset are a byproduct of a decrease in motor preparation after response execution. (b) Response-locked motor preparation has a negative relationship with RT until around 300 ms before the response, reflecting the fact that motor preparation builds over a narrower timeframe on the trials with faster RT, and then reaches uniform levels at the time of response execution. (c) Stimulus-locked CPP predicts RT between 200 and 600 ms after evidence onset, consistent with shallower evidence integration on trials with slower responses. Similar to motor preparation, positive correlations between CPP amplitude and RT beyond about 700 ms result from the decrease in signal amplitude once a response is made. (d) As expected, response-locked CPP traces show positive correlations with RT well in advance of the response and negative correlations after response commitment. Replicating the results of trials split by condition (Figure 4h), CPP amplitude is lower for very fast and very slow responses around the time of decision commitment (-130 to -70 ms). (e)

Stimulus-locked SSVEP does not predict RT at any time point before the mean reaction time of the fastest RT bin. (f) Response-locked SSVEP amplitude correlates positively with RT until 350 ms before the response. This is simply due to the initial ramp-up of the SSVEP differential at evidence onset occurring at different pre-response times for the different RT bins.

Supplementary Methods

Statistical analysis details

As described in the Statistical Analysis section of the Methods, we used linear mixed-effects models to statistically test effects that were expected to depend on reaction time. All linear mixed-effects models were constructed to include the fixed effect factors of reaction time (RT), RT^2, Regime (Speed/Accuracy), stimulus Contrast, and Trial Type (Left/Right) to maintain consistency across measures. Linear mixed-effects (LME) models were constructed to include random slopes factors that significantly contribute to goodness-of-fit. This was determined through an iterative procedure, where each random slopes factor was tested in turn using chi-squared tests. Below we exhaustively list the steps in this model construction for each of the LME tests carried out in the paper. The upper part (white background) of each table shows the tests for inclusion of each individual random slopes factor to construct the full model, and the bottom part (gray background) gives the chi-squared tests for each fixed-effect factor in the final model, as quoted in the main text.

Supplementary Table 1: Results of linear mixed-effects models

a — Mu/Beta amplitude at baseline (executed)

Baseline model

Mu/Beta baseline ~ RT + RT^2 + Speed/Accuracy + Contrast + Left/Right + (1 | Subject)

Random slope	χ^2	DF	p
RT	18.97	2	0.0000759
RT^2	14.23	2	0.00081
Speed/Accuracy	1.19	2	0.55
Left/Right	0	2	1
Contrast	0	2	1

Final model

Mu/Beta baseline ~ RT + RT^2 + Speed/Accuracy + Contrast + Left/Right + (1 + RT + RT^2 | Subject)

Effect	χ^2	DF	p
RT	10.09	1	0.0015
RT^2	5.9	1	0.015
Speed/Accuracy	11.91	1	0.00056
Left/Right	0.00012	1	0.99
Contrast	3.12	1	0.077

b — Mu/Beta amplitude at baseline (withheld)

Baseline model

Mu/Beta baseline ~ RT + RT^2 + Speed/Accuracy + Contrast + Left/Right + (1 | Subject)

Random slope	χ^2	DF	p
RT	7.71	2	0.021
RT^2	7.50	2	0.024
Speed/Accuracy	2.29	2	0.32
Left/Right	0	2	1
Contrast	$7.28*10^{-12}$	2	1

Final model

Mu/Beta baseline ~ RT + RT^2 + Speed/Accuracy + Contrast + Left/Right + (1 + RT + RT^2 | Subject)

Effect	χ^2	DF	p
RT	3.91	1	0.048
RT^2	1.31	1	0.25
Speed/Accuracy	16.25	1	$5.55*10^{-5}$
Left/Right	0.11	1	0.74
Contrast	1.42	1	0.23

c — Mu/Beta amplitude at response (executed)

Baseline model

Mu/Beta at RT ~ RT + RT^2 + Speed/Accuracy + Contrast + Left/Right + (1 | Subject)

Random slope	χ^2	DF	p
RT	35.67	2	$1.80*10^{-8}$
RT^2	22.56	2	0.0000126
Speed/Accuracy	18.19	2	0.00011
Left/Right	17.79	2	0.00014
Contrast	25.15	2	$3.45*10^{-6}$

Final model

Mu/Beta at RT ~ RT + RT^2 + Speed/Accuracy + Contrast + Left/Right + (1 + RT + RT^2 + Speed/Accuracy + Contrast + Left/Right | Subject)

Effect	χ^2	DF	p
RT	0.42	1	0.52
RT^2	0.026	1	0.87
Speed/Accuracy	0.55	1	0.46
Left/Right	0.72	1	0.40
Contrast	1.93	1	0.17

d — CPP amplitude at baseline

Baseline model

CPP baseline ~ RT + RT^2 + Speed/Accuracy + Contrast + Left/Right + (1 | Subject)

Random slope	χ^2	DF	p
RT	$4.37*10^{-11}$	2	1
RT^2	0.77	2	0.68
Speed/Accuracy	$1.16*10^{-10}$	2	1
Left/Right	0.92	2	0.63
Contrast	2.32	2	0.31

Final model

CPP baseline ~ RT + RT^2 + Speed/Accuracy + Contrast + Left/Right + (1 | Subject)

Effect	χ^2	DF	p
RT	0.89	1	0.35
RT^2	0.012	1	0.91
Speed/Accuracy	1.51	1	0.22
Left/Right	0.0013	1	0.97
Contrast	0.042	1	0.84

Supplementary Table 2: Results of linear mixed-effects models (continued)

a	CPP amplitude at response	Baseline model	**CPP at RT ~ RT + RT^2 + Speed/Accuracy + Contrast + Left/Right + (1 | Subject)**			
			Random slope	χ^2	DF	
			RT	18.97	2	0.0000759
			RT^2	14.23	2	0.00081
			Speed/Accuracy	1.19	2	0.55
			Left/Right	0	2	1
			Contrast	0	2	1
		Final model	**CPP at RT ~ RT + RT^2 + Speed/Accuracy + Contrast + Left/Right + (1 + Contrast | Subject)**			
			Effect	χ^2	DF	
			RT	10.09	1	0.0015
			RT^2	5.9	1	0.015
			Speed/Accuracy	11.91	1	0.00056
			Left/Right	0.00012	1	0.99
			Contrast	3.12	1	0.077
b	EMG rate of rise (executed)	Baseline model	**EMG slope ~ RT + RT^2 + Speed/Accuracy + Contrast + Left/Right + (1 | Subject)**			
			Random slope	χ^2	DF	
			RT	7.71	2	0.021
			RT^2	7.50	2	0.024
			Speed/Accuracy	2.29	2	0.32
			Left/Right	0	2	1
			Contrast	$7.28*10^{-12}$	2	1
		Final model	**EMG slope ~ RT + RT^2 + Speed/Accuracy + Contrast + Left/Right + (1 + RT + RT^2 + Speed/Accuracy + Contrast + Left/Right | Subject)**			
			Effect	χ^2	DF	
			RT	3.91	1	0.048
			RT^2	1.31	1	0.25
			Speed/Accuracy	16.25	1	$5.55*10^{-5}$
			Left/Right	0.11	1	0.74
			Contrast	1.42	1	0.23
c	EMG amplitude at response (executed)	Baseline model	**EMG at RT ~ RT + RT^2 + Speed/Accuracy + Contrast + Left/Right + (1 | Subject)**			
			Random slope	χ^2	DF	
			RT	35.67	2	$1.80*10^{-8}$
			RT^2	22.56	2	0.0000126
			Speed/Accuracy	18.19	2	0.00011
			Left/Right	17.79	2	0.00014
			Contrast	25.15	2	$3.45*10^{-6}$
		Final model	**EMG at RT ~ RT + RT^2 + Speed/Accuracy + Contrast + Left/Right + (1 + RT + RT^2 + Speed/Accuracy + Contrast + Left/Right | Subject)**			
			Effect	χ^2	DF	
			RT	0.42	1	0.52
			RT^2	0.026	1	0.87
			Speed/Accuracy	0.55	1	0.46
			Left/Right	0.72	1	0.40
			Contrast	1.93	1	0.17
d	EMG amplitude at response (withheld)	Baseline model	**EMG at RT ~ RT + RT^2 + Speed/Accuracy + Contrast + Left/Right + (1 | Subject)**			
			Random slope	χ^2	DF	
			RT	$4.37*10^{-11}$	2	1
			RT^2	0.77	2	0.68
			Speed/Accuracy	$1.16*10^{-10}$	2	1
			Left/Right	0.92	2	0.63
			Contrast	2.32	2	0.31
		Final model	**EMG at RT ~ RT + RT^2 + Speed/Accuracy + Contrast + Left/Right + (1 + RT + RT^2 + Speed/Accuracy + Left/Right | Subject)**			
			Effect	χ^2	DF	
			RT	0.89	1	0.35
			RT^2	0.012	1	0.91
			Speed/Accuracy	1.51	1	0.22
			Left/Right	0.0013	1	0.97
			Contrast	0.042	1	0.84

Supplementary Table 3: Results of linear mixed-effects models (continued)

a	Mu/Beta "excursion" (withheld)	Baseline model	**Mu/Beta exc. ~ RT + RT^2 + Speed/Accuracy + Contrast + Left/Right + (1 | Subject)**			
			Random slope	χ^2	DF	p
			RT	11.46	2	0.033
			RT^2	0.038	2	0.98
			Speed/Accuracy	11.29	2	0.0035
			Left/Right	71.91	2	$2.22*10^{-16}$
			Contrast	1.96	2	0.37
		Final model	**Mu/Beta exc. ~ RT + RT^2 + Speed/Accuracy + Contrast + Left/Right + (1 + RT + Speed/Accuracy + Left/Right | Subject)**			
			Effect	χ^2	DF	p
			RT	2.76	1	0.097
			RT^2	0.82	1	0.37
			Speed/Accuracy	2.31	1	0.13
			Left/Right	8.11	1	0.0044
			Contrast	2.36	1	0.12
b	CPP amplitude at response (with AEP)	Baseline model	**CPP at RT ~ RT + RT^2 + Speed/Accuracy + Contrast + Left/Right + (1 | Subject)**			
			Random slope	χ^2	DF	p
			RT	27.63	2	$1.00*10^{-6}$
			RT^2	2.35	2	0.31
			Speed/Accuracy	4.21	2	0.12
			Left/Right	6.77	2	0.034
			Contrast	13.28	2	0.0013
		Final model	**CPP at RT ~ RT + RT^2 + Speed/Accuracy + Contrast + Left/Right + (1 + RT + Contrast + Left/Right | Subject)**			
			Effect	χ^2	DF	p
			RT	23.22	1	$1.45*10^{-6}$
			RT^2	1.45	1	0.23
			Speed/Accuracy	4.22	1	0.040
			Left/Right	1.18	1	0.28
			Contrast	2.34	1	0.13
c	Mu/Beta baseline difference (exec. – with.)	Baseline model	**Mu/Beta base. diff. ~ RT + RT^2 + Speed/Accuracy + Contrast + Left/Right + (1 | Subject)**			
			Random slope	χ^2	DF	p
			RT	1.75	2	0.42
			RT^2	0.33	2	0.85
			Speed/Accuracy	$-7.28*10^{-12}$	2	1
			Left/Right	$-7.28*10^{-12}$	2	1
			Contrast	0.021	2	0.99
		Final model	**Mu/Beta baseline diff. ~ RT + RT^2 + Speed/Accuracy + Contrast + Left/Right + (1 | Subject)**			
			Effect	χ^2	DF	p
			RT	4.65	1	0.031
			RT^2	3.23	1	0.072
			Speed/Accuracy	0.30	1	0.58
			Left/Right	0.039	1	0.84
			Contrast	0.20	1	0.65

Supplementary Table 4: Full results of the ANOVAs reported in the main text

This table lists the results of all Analyses of Variance reported in this study. An alpha level of 0.05 was set for all tests. For details regarding the direction and interpretation of these effects, please refer to the Results and Supplementary Information.

AEP = auditory evoked potential; CAF = conditional accuracy function; CPP = centro-parietal positivity; SSVEP = Steady-State Visual Evoked Potential.

			F	df1	df2	p
a	Reaction time	Speed/Accuracy	46.63	1	15	$5.71*10^{-6}$
		Contrast	86.67	1	15	$1.27*10^{-7}$
		Speed/Accuracy * Contrast	23.47	1	15	0.00021455
b	Response accuracy	Speed/Accuracy	23.18	1	15	0.00022753
		Contrast	106.81	1	15	$3.23*10^{-8}$
		Speed/Accuracy * Contrast	3.86	1	15	0.068
c	Decline in CAF (6 bins for cut-off)	Speed/Accuracy	1.66	1	15	0.22
		Contrast	2.70	1	15	0.12
		Left/Right	0.021	1	15	0.89
		Speed/Accuracy * Contrast	1.16	1	15	0.30
		Speed/Accuracy * Left/Right	0.39	1	15	0.54
		Contrast * Left/Right	0.073	1	15	0.79
		Speed/Accuracy * Contrast * Left/Right	1.08	1	15	0.32
d	Stimulus-locked SSVEP amplitude	Speed/Accuracy	0.047	1	15	0.83
		Contrast	3.52	1	15	0.080
		Left/Right	26.77	1	15	0.0028
		Speed/Accuracy * Contrast	0.99	1	15	0.34
		Speed/Accuracy * Left/Right	5.47	1	15	0.034
		Contrast * Left/Right	41.24	1	15	$1.15*10^{-5}$
		Speed/Accuracy * Contrast * Left/Right	1.79	1	15	0.20
e	Response-locked SSVEP (-50ms)	Speed/Accuracy	$1.07*10^{-7}$	1	15	1.00
		Contrast	2.32	1	15	0.15
		Left/Right	27.28	1	15	0.0026
		Speed/Accuracy * Contrast	0.014	1	15	0.91
		Speed/Accuracy * Left/Right	5.67	1	15	0.031
		Contrast * Left/Right	38.01	1	15	$1.81*10^{-5}$
		Speed/Accuracy * Contrast * Left/Right	1.28	1	15	0.28
f	Response-locked SSVEP (+50ms)	Speed/Accuracy	0.28	1	15	0.61
		Contrast	1.79	1	15	0.20
		Left/Right	26.50	1	15	0.0082
		Speed/Accuracy * Contrast	4.64	1	15	0.048
		Speed/Accuracy * Left/Right	2.90	1	15	0.11
		Contrast * Left/Right	41.46	1	15	$1.12*10^{-5}$
		Speed/Accuracy * Contrast * Left/Right	2.58	1	15	0.13
g	Stimulus-locked SSVEP (20 Hz)	Speed/Accuracy	0.36	1	15	0.56
		Contrast	24.26	1	15	0.00018
		Left/Right	8.98	1	15	0.0090
		Speed/Accuracy * Contrast	2.39	1	15	0.14
		Speed/Accuracy * Left/Right	0.53	1	15	0.48
		Contrast * Left/Right	8.65	1	15	0.010
		Speed/Accuracy * Contrast * Left/Right	1.94	1	15	0.18
h	Stimulus-locked SSVEP (25Hz)	Speed/Accuracy	2.49	1	15	0.14
		Contrast	1.60	1	15	0.23
		Left/Right	21.94	1	15	0.00029
		Speed/Accuracy * Contrast	0.72	1	15	0.41
		Speed/Accuracy * Left/Right	1.97	1	15	0.18
		Contrast * Left/Right	23.58	1	15	0.00021
		Speed/Accuracy * Contrast * Left/Right	0.75	1	15	0.40
i	Stimulus-locked SSVEP (correct vs. incorrect)	Correct/Incorrect	0.38	1	15	0.55
		Left/Right	21.49	1	15	0.00032
		Speed/Accuracy	0.029	1	15	0.87
		Correct/Incorrect * Left/Right	7.71	1	15	0.014
		Correct/Incorrect * Speed/Accuracy	0.036	1	15	0.85
		Left/Right * Speed/Accuracy	4.62	1	15	0.048
		Correct/Incorrect * Left/Right * Speed/Accuracy	0.073	1	15	0.79

Supplementary Table 5: Full results of the ANOVAs reported in the main text (continued)
This table lists the results of all Analyses of Variance reported in this study. An alpha level of 0.05 was set for all tests. For details regarding the direction and interpretation of these effects, please refer to the Results and Supplementary Information.
AEP = auditory evoked potential; CAF = conditional accuracy function; CPP = centro-parietal positivity; SSVEP = Steady-State Visual Evoked Potential.

			F	df1	df2	p
**a**	**Pupil diameter over time**	Speed/Accuracy	23.55	29	435	0.031
		Time	41.92	29	435	$2.17*10^{-6}$
		Speed/Accuracy * Time	17.62	29	435	0.00046
**b**	**Pupil and SSVEP**	Speed/Accuracy	0.038	1	15	0.85
		Contrast	2.78	1	15	0.12
		Left/Right	27.57	1	15	0.0026
		Pupil diameter	1.12	1	15	0.31
		Speed/Accuracy * Contrast	0.31	1	15	0.59
		Speed/Accuracy * Left/Right	10.71	1	15	0.0051
		Speed/Accuracy * Pupil diameter	0.092	1	15	0.77
		Contrast * Left/Right	39.68	1	15	$1.43*10^{-5}$
		Contrast * Pupil diameter	1.17	1	15	0.30
		Left/Right * Pupil diameter	10.83	1	15	0.0050
		Speed/Accuracy * Contrast * Left/Right	1.32	1	15	0.27
		Speed/Accuracy * Contrast * Pupil	0.12	1	15	0.73
		Speed/Accuracy * Left/Right * Contrast	3.10	1	15	0.098
		Contrast * Left/Right * Pupil diameter	1.29	1	15	0.27
		Speed/Accuracy * Contrast * Left/Right * Pupil	1.73	1	15	0.21
**c**	**Mu/Beta rate of rise (executed response)**	Speed/Accuracy	11.51	1	15	0.004
		Contrast	9.22	1	15	0.0083
		Speed/Accuracy * Contrast	0.58	1	15	0.46
**d**	**CPP rate of rise**	Speed/Accuracy	5.43	1	15	0.034
		Contrast	12.99	1	15	0.0026
		Speed/Accuracy * Contrast	0.23	1	15	0.64
**e**	**Mu/Beta amplitude at response**	Speed/Accuracy	3.08	1	15	1.00
		Contrast	0.036	1	15	0.85
		Executed/Withheld	14.26	1	15	0.0018
		Speed/Accuracy * Contrast	0.26	1	15	0.62
		Speed/Accuracy * Executed/Withheld	0.00014	1	15	0.99
		Contrast * Executed/Withheld	2.30	1	15	0.15
		Speed/Accuracy * Contrast * Executed/Withheld	1.75	1	15	0.21
**f**	**Response-locked CPP peak time**	Speed/Accuracy	14.04	1	15	0.0019
		Contrast	0.32	1	15	0.58
		Speed/Accuracy * Contrast	1.85	1	15	0.19
**g**	**Difference CPP peak - motor time**	Speed/Accuracy	7.83	1	15	0.014
		Contrast	0.0061	1	15	0.94
		Speed/Accuracy * Contrast	2.07	1	15	0.17
**h**	**Points won**	Speed/Accuracy	68.85	1	15	$5.48*10^{-7}$
		Deadline/Slope	4.49	1	15	0.051
		Speed/Accuracy * Deadline/Slope	64.45	1	15	$8.24*10^{-7}$
**i**	**CPP rate of rise (with AEP)**	Speed/Accuracy	11.64	1	15	0.0039
		Contrast	21.00	1	15	0.00036
		Speed/Accuracy * Contrast	0.16	1	15	0.70

***Supplementary** References*

1. *O'Connell, R. G., Dockree, P. M. & Kelly, S. P. A supramodal accumulation-to-bound signal that determines perceptual decisions in humans. Nat. Neurosci.* ***15,*** *1729–1735 (2012).*
2. *Dmochowski, J. P. & Norcia, A. M. Cortical Components of Reaction-Time during Perceptual Decisions in Humans. PLOS ONE* ***10,*** *e0143339 (2015).*

3. *Roitman, J. D. & Shadlen, M. N. Response of Neurons in the Lateral Intraparietal Area during a Combined Visual Discrimination Reaction Time Task. J. Neurosci.* ***22,*** *9475–9489 (2002).*
